# Quantitative expression of MMPs 2, 9, 14, and collagen IV in LCIS and paired normal breast tissue

**DOI:** 10.1038/s41598-019-48602-6

**Published:** 2019-09-17

**Authors:** Sarah J. Nyante, Tengteng Wang, Xianming Tan, Emily F. Ozdowski, Thomas J. Lawton

**Affiliations:** 1 Department of Radiology, University of North Carolina at Chapel Hill, Chapel Hill, NC USA; 2 Department of Epidemiology, University of North Carolina at Chapel Hill, Chapel Hill, NC USA; 3 Lineberger Comprehensive Cancer Center, University of North Carolina at Chapel Hill, Chapel Hill, NC USA; 4 Department of Biostatistics, University of North Carolina at Chapel Hill, Chapel Hill, NC USA; 5 Department of Pathology and Laboratory Medicine, University of California, Los Angeles, Los Angeles, CA USA

**Keywords:** Breast cancer, Cancer microenvironment, Cancer prevention

## Abstract

Certain matrix metalloproteinases (MMPs) have the ability to degrade collagen IV, a main component of the breast lobular basement membrane. In this cross-sectional study, we evaluated expression of MMPs 2, 9, and 14 and collagen IV in LCIS and adjacent normal breast tissue among LCIS patients without invasive breast cancer to determine whether expression differed between benign and preinvasive breast epithelial tissue. A total of 64 LCIS patients, diagnosed 2004–2014, were included; 44 had sufficient paired normal tissue for analysis. Marker epithelial expression was measured using immunofluorescence and quantified using the H score (MMPs) or pixel intensity (collagen IV). Associations were evaluated using the Spearman correlation or the Wilcoxon signed-rank test. In LCIS and normal tissue, there was a strong correlation between MMP2 and MMP14 expression (LCIS r = 0.69, normal r = 0.81, both P < 0.01). Other pairwise correlations were moderate to weak (range: LCIS r = 0.32–0.47, normal r = 0.19–0.32). For all markers, expression was lower in LCIS vs. normal tissue (all P ≤ 0.05). In sum, collagenase MMPs were expressed in normal breast and LCIS lesions of LCIS patients. However, expression was not higher in LCIS compared with normal tissue, suggesting collagenase MMP expression does not increase as breast tissue gains a more proliferative phenotype.

## Introduction

Women with lobular carcinoma *in situ* (LCIS) have an estimated 6 to 11-fold increased risk of invasive breast cancer compared with the general population^1–3^. Although LCIS has long been considered as a marker of increased breast cancer risk, some in the literature have proposed that a subset of LCIS may also be non-obligatory precursors of invasive breast cancer^4,5^. These assertions are supported by a growing body of research, which shows that LCIS and invasive lobular cancer share a unique pattern of genetic mutations and progressive alterations in gene expression^6–10^. However, the specific molecular factors leading to the increased risk of invasive breast cancer in LCIS patients remain largely unknown.

Epithelial cell invasion into the stroma is thought to require degradation of the basement membrane^11^. Though rare, there is histologic evidence that LCIS epithelial cells have the ability to break through the basement membrane and directly invade the breast stroma^12–15^. In breast cancer, one class of molecules implicated in this type of degradation are matrix metalloproteinases (MMPs)^16,17^. MMPs 2 and 9 cleave collagen IV, a key component of the breast glandular basement membrane^16,17^. MMP14 is an activator of MMP2 and may be an important determinant of its collagenase activity^18^. Prior studies have demonstrated that MMPs 2, 9, and 14 are expressed in 70–100% of invasive breast tumors, with intense expression in approximately 50% of cells^18–29^. However, the degree to which MMPs 2, 9, and 14 are expressed prior to invasion is unknown.

Much of the existing data regarding MMP expression in LCIS and normal breast tissue comes from convenience samples obtained from patients with invasive breast cancer. Given the known ability of invasive tumors to influence host tissue, it is unclear whether MMP expression in those LCIS lesions precedes the invasive tumor, or if expression levels are a result of the influence of the tumor. To address this question, we conducted a pilot study to estimate quantitative expression of MMP2, MMP9, MMP14, and collagen IV in archival tissues from women diagnosed with LCIS without invasive breast cancer. We compared quantitative expression patterns between LCIS and adjacent normal breast tissue samples from the same patient in order to identify markers of altered lobular tissue morphology. The results from this study will clarify our understanding of the biological pathways that may play a role in the development of invasive breast carcinoma among LCIS patients.

## Results

### Population characteristics

This analysis included data from 64 patients with LCIS and no synchronous invasive breast cancer. A majority were diagnosed with the ‘classic’ histologic subtype of LCIS. Patient characteristics are shown in Supplementary Table S1. Briefly, the majority of women were white, postmenopausal, and had dense breasts. The mean patient age was 53 years (range 32 to 76).

### Quantification of MMP2, MMP9, MMP14, and collagen IV expression in LCIS

**Figure 1:**
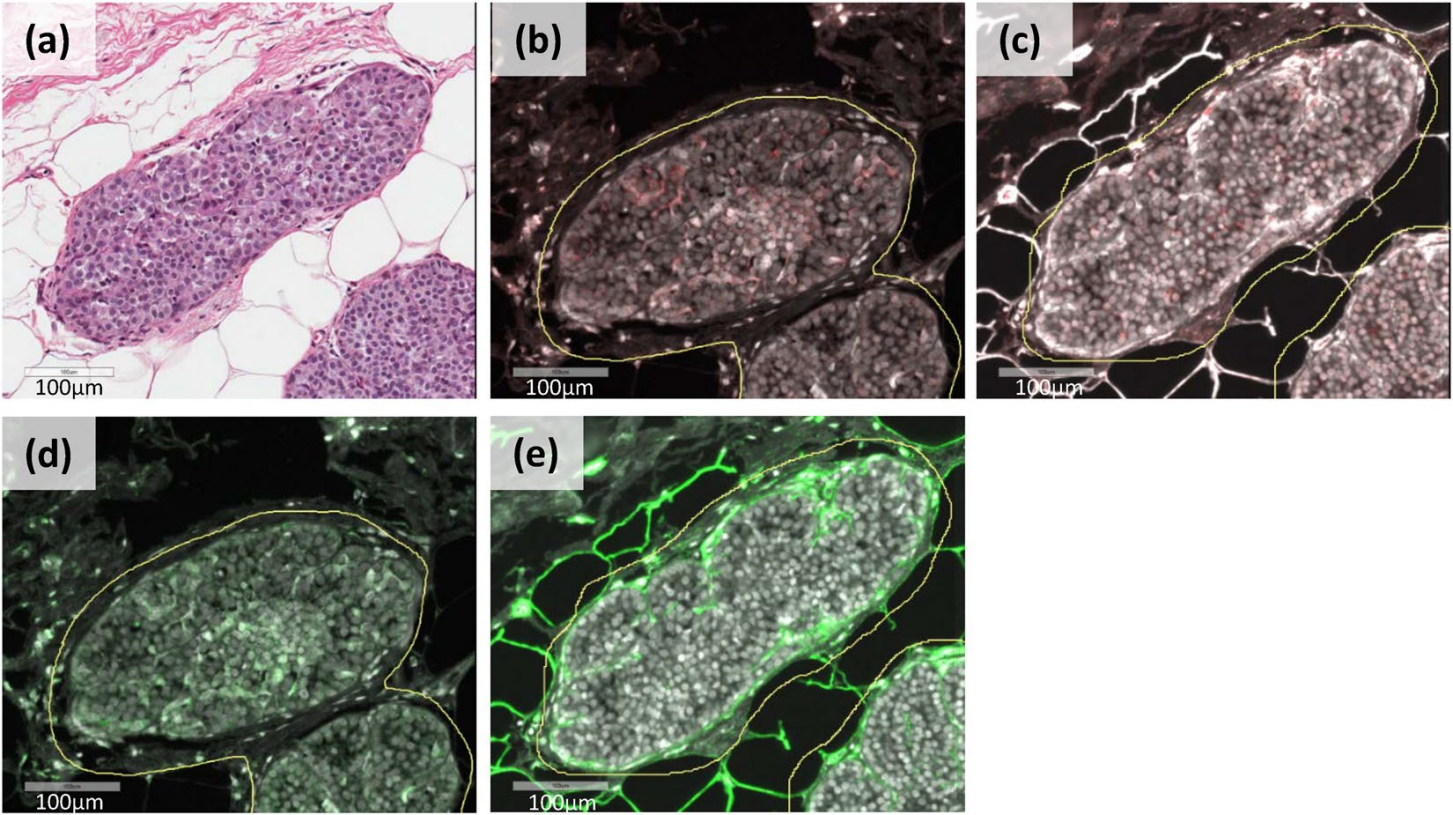
Scanned images of a representative area of LCIS from a single case. The H&E-stained image (panel a) was used as a guide to mark the areas of epithelial tissue for analysis (annotations shown in yellow). Markers were stained using dual-immunofluorescence. MMP2 (panel b) and MMP9 (panel c) were visualized with Cy5 (red), whereas MMP14 (panel d) and collagen IV (panel e) were visualized with Cy3 (green). The images are shown at 20x magnification.

**Figure 2:**
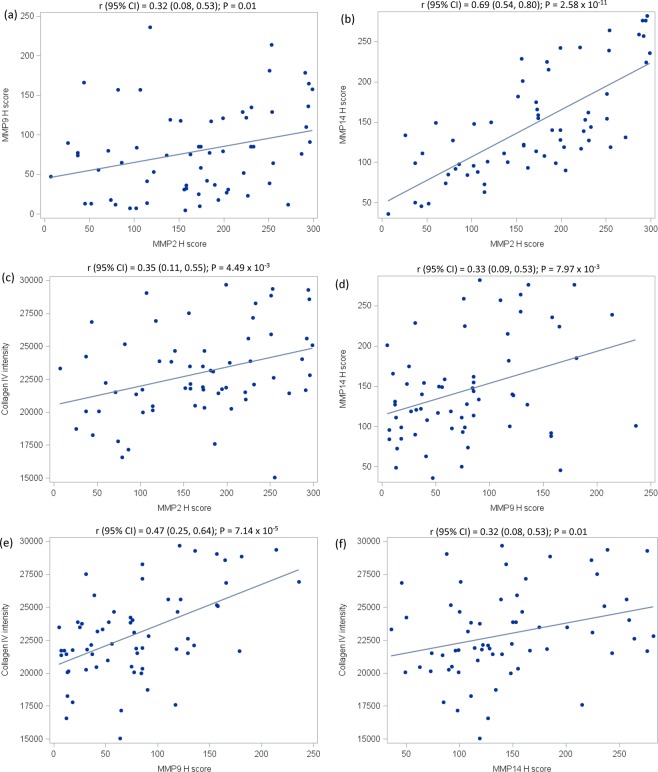
Correlation between quantitative expression of MMP2, MMP9, MMP14, and collagen IV in LCIS among 64 cases diagnosed at a single institution, 2004–2014. Panels a-f show pairwise relationships between expression of MMPs 2, 9, and 14 and collagen IV. The associated Spearman correlation and 95% confidence interval are shown directly above each panel. The solid line shown in each plot represents the linear regression model-based fit line.

MMP2, MMP9, MMP14, and collagen IV expression was evaluated using dual immunofluorescence staining on formalin-fixed paraffin-embedded tissue (Fig. 1). MMP2, MMP9, and MMP14 expression was common in LCIS: 98% of samples expressed MMP2, 86% expressed MMP9, and 100% expressed MMP14. Most samples (85%) expressed all three markers; 14% expressed only MMP2 and MMP14; and 2% expressed only MMP9 and MMP14 (Supplementary Data). When considering the extent and intensity of expression, as measured by the H Score, there was a strong correlation between MMP2 and MMP14 expression (r = 0.69, 95% CI 0.54–0.80; Fig. 2). Correlations between expression levels of MMP2 and MMP9 (r = 0.32, 95% CI 0.08–0.53) and MMP9 and MMP14 (r = 0.33, 95% CI 0.09–0.53) were moderate (Fig. 2). There was a moderate correlation between collagen IV expression and all three MMPs in LCIS (Fig. 2). The distribution of expression for each marker did not differ according to LCIS histologic subtype (Supplementary Fig. S1).

### Quantification of MMP2, MMP9, MMP14, and collagen IV expression in normal breast tissue

**Figure 3:**
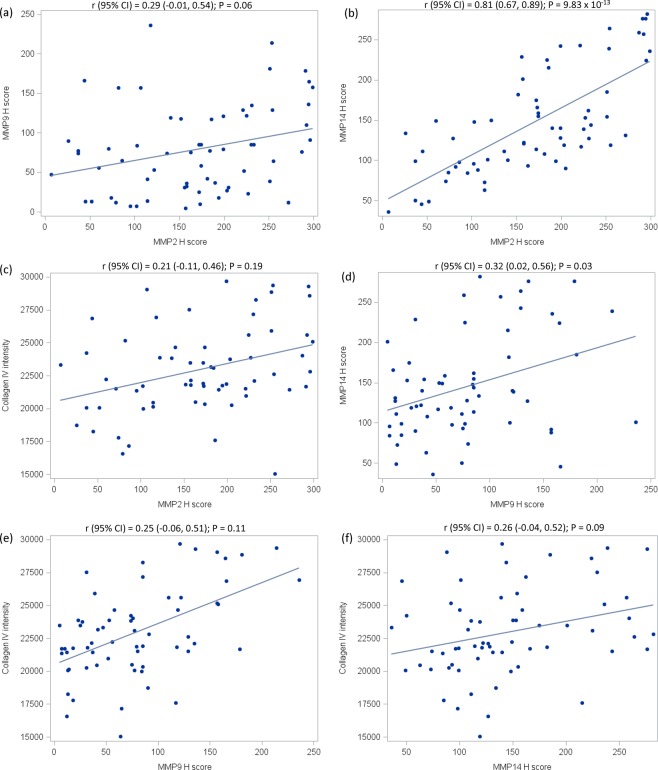
Correlation between quantitative expression of MMP2, MMP9, MMP14, and collagen IV in 44 samples of normal breast epithelium from women diagnosed with LCIS at a single institution, 2004–2014. Panels a-f show pairwise relationships between expression of the MMPs 2, 9, and 14 and collagen IV. The associated Spearman correlation and 95% confidence interval are shown directly above each panel. The solid line shown in each plot represents the linear regression model-based fit line.

Of the 64 LCIS patients, 44 had sufficient paired normal tissue for comparison. Cases with paired normal tissue did not differ from those without paired normal tissue with respect to LCIS marker expression or most patient characteristics, but tended to be younger and were more likely to have LCIS diagnosed from a surgical biopsy or mastectomy rather than a needle-core biopsy (Supplementary Table S1 and Supplementary Fig. S2). MMP2 and MMP14 were expressed in 100% of the normal samples and MMP9 was expressed in 98% of the normal samples (Supplementary Data). Similar to the LCIS samples, there was a strong correlation between MMP2 and MMP14 expression (r = 0.81, 95% CI 0.67–0.89), and moderate correlations between MMPs 2 and 9 (r = 0.29, 95% CI −0.01–0.54) and MMPs 9 and 14 (r = 0.32, 95% CI 0.02–0.56) (Fig. 3). MMP correlations with collagen IV were moderate to weak (Fig. 3).

### Comparison of MMP and collagen IV expression in LCIS and paired normal breast tissue

For all MMPs, H scores were consistently lower in LCIS when compared with normal breast epithelial tissue (all P < 0.01, Table 1). Median collagen IV expression was higher in normal breast tissue compared with LCIS; the difference was of borderline statistical significance (normal: median intensity 23313, interquartile range: 20799–25499; LCIS median intensity 22211, interquartile range: 20966–25097) (Wilcoxon signed-rank test P = 0.05).

**Table 1:** Comparison of quantitative H score expression in LCIS and normal breast epithelium for MMP2, MMP9, and MMP14 in 44 cases of LCIS with available paired normal tissue, 2004–2014.

Marker	H Score Median (interquartile range)		P-value^a^
	LCIS	Paired normal breast	
MMP 2	178 (116–226)	218 (137–257)	9.30 × 10^−3^
MMP 9^b^	77 (36–121)	102 (67–156)	8.62 × 10^−3^
MMP 14	140 (105–174)	171 (118–223)	7.25 × 10^−3^

^a^Wilcoxon signed-rank test comparing H score distribution between paired LCIS and normal.

^b^Missing data: MMP9 – 1 observation.

## Discussion

It has been proposed that some LCIS are non-obligatory precursors of invasive lobular breast cancer^4,5^, suggesting that the biological determinants of breast cancer are present at the *in situ* stage. However, a lack of understanding of the molecular mechanisms linking LCIS to invasive breast cancer has slowed acceptance of the precursor hypothesis. Our investigation was based on a framework for pre-malignant lesion progression, in which cellular changes and the microenvironment were identified as major mechanisms likely responsible for increased breast cancer risk^4^. MMPs 2, 9, and 14 are instrumental in the breakdown of collagen IV, a principal structural component of the breast extracellular matrix, and are therefore strong candidates for involvement in LCIS progression^16,17^.

In this cross-sectional study, we examined expression levels of MMPs 2, 9, and 14 and collagen IV in 64 women diagnosed with LCIS to ascertain LCIS MMP expression levels in the absence of invasive breast cancer. We also examined expression in normal breast glandular epithelium in these same patients to determine whether MMP or collagen IV expression in the *in situ* lesions differed in comparison to phenotypically normal glands. We found that MMP 2, 9, and 14 expression was common in LCIS and normal breast tissue of LCIS patients. Unexpectedly, there was a positive correlation between MMP expression and collagen IV expression, though collagen IV levels were slightly lower in LCIS as compared with normal breast samples from the same woman.

Most prior studies of MMPs 2, 9, and 14 in breast cancer have reported MMP expression in breast tumor cells (which are epithelial in origin); some also have reported stromal expression, but at weaker levels than tumor expression^19,21–23,26,27,30–32^. Some of these same studies reported that MMPs 2, 9, and 14 were expressed in normal, benign, and *in situ* tissues, with most^19,22,26,28,32–34^, but not all^35^, reporting epithelial expression. The few studies to examine LCIS specifically provided evidence that MMPs 2 and 9 were expressed, but sample sizes were either very small or not reported (MMP2–5 samples^35^ and 10 samples^33^; MMP9–20 samples^33^ and 1 sample^19^), making the generalizability of the data questionable. To our knowledge, there is no published data on the expression of MMP14 in LCIS. Our study of 64 well-characterized samples, systematically obtained from an institutional case series, confirms the expression of these markers in LCIS. The comparatively large size of our case group in addition to our method of case selection suggest that our findings will be generalizable to LCIS diagnosed in other contexts. Moreover, our results showing that MMPs 2, 9, and 14 are expressed in LCIS support the hypothesis that there may be MMP activity at the *in situ* stage that contributes to the development of invasive breast cancer.

Few studies have quantified levels of MMP staining observed in non-malignant tissue. MMP2 is the only marker for which more than one prior study reported the level of expression, and results were inconsistent. One study^22^ described MMP2 staining in normal tissue and the intraepithelial neoplastic component as “weak”; a second study^35^ described MMP2 staining as “weak” in normal tissue and “moderate” in LCIS; a third study^19^ reported “strong” MMP2 reactivity around ducts and some acini in normal and benign tissue. Our study’s finding that MMP2 expression was in the upper end of the H score range for LCIS and normal tissue is consistent with reported moderate staining in LCIS and strong staining in normal and benign tissue. However, subjective terms such as “weak,” “moderate,” and “strong” are difficult to interpret without a known point of reference. The use of an objective scoring scale, such as the H score, is needed to ensure that results can be compared across studies.

The factors influencing MMP expression in LCIS and normal breast epithelium remain unknown. In this study, we chose to compare LCIS expression with paired normal tissue from the same breast to understand how LCIS MMP expression differs from “background” levels in the same breast, an approach that controls for patient-level characteristics. However, the use of other types of normal comparison tissue, such as tissue from the contralateral breast or from women unaffected by pre-neoplastic changes, may yield different results. MMP expression levels in the normal tissue of LCIS patients may differ from MMP expression in women without any evidence of abnormal tissue growth. Thus, it remains to be determined whether the levels of MMP and collagen IV expression observed in the normal tissues in this study are generalizable to women without LCIS. Future studies evaluating differences in MMP expression between LCIS patients and women with no known pre-neoplastic breast changes will be informative for understanding these differences.

There are several limitations that may affect the interpretation of our findings. Not all LCIS samples had paired normal tissue, resulting in limited statistical power for analyses of paired LCIS and normal tissues. Due to the lack of existing quantitative expression data for these markers in LCIS or normal breast tissue, the power calculations used to determine the study sample size were based on estimated data. *Post hoc* power calculations showed that we had greater than 80% power to detect a difference in the distributions of MMP9 and MMP14 in LCIS and normal tissue. Power was less than 80% for the analysis of MMP2; thus, those results should be interpreted with caution. Additionally, women with paired normal tissue differed from women without paired normal tissue according to some personal characteristics. However, it is unclear what, if any, effect this may have had on our results, given that marker expression in LCIS did not differ between cases with and without paired normal tissue. We did not have information on the distance between paired LCIS and normal epithelial lesions and were unable to examine the effect of between-lesion distance on differences in marker expression. Furthermore, our assessment of marker expression was unable to distinguish whether the MMPs were in their active or inactive form. Analyses using alternative molecular approaches that quantify active MMP levels are needed to further understand the relationship between MMP and collagen IV levels in LCIS. Our automated tissue segmentation method did not separate myoepithelial cells from other epithelial cells, which prevented us from being able to determine the contributions of different epithelial cell types to overall glandular expression levels. Some studies suggest that MMP 2, 9, and 14 expression is limited to myoepithelial cells in normal lobules, as might be expected for breast development^19,26,28,33,35^. However, our use of careful manual annotation ensured that MMP expression in cell types outside the lobular unit, such as fibroblasts, was excluded from analysis.

Despite these limitations, the study has several strengths that complement prior research in this area. This study of 64 LCIS is the largest case series to examine expression of these MMPs and collagen IV in the absence of synchronous invasive breast cancer. Measurement of MMP expression in this population ensured that observed expression was not influenced by the presence of an adjacent invasive carcinoma. All cases and annotations were reviewed by an experienced pathologist with a specialization in breast pathology to verify the LCIS diagnosis. We used an automated, validated digital scoring algorithm in commercially-available software to measure MMP expression with a quantitative scale (H score). Such an automated scoring method reduces the potential for within and across study measurement variability that can result from subjective scoring methods.

In summary, despite numerous studies characterizing MMPs 2, 9, and 14 expression in invasive breast tumors^19–29,36,37^, there was little prior data quantifying expression of these factors prior to tumor development. We purposefully undertook this pilot study of MMP and collagen IV levels in pure LCIS to establish expression levels of these MMPs in a well-characterized sample of LCIS patients. Our results demonstrate that MMPs associated with collagenase activity are expressed in normal and LCIS breast epithelial cells among patients without synchronous invasive breast cancer. However, MMP expression was not inversely correlated with collagen IV expression. Due to this study’s cross-sectional design, our data do not address whether there is a causal relationship between the level of MMP expression in LCIS and the level of expression in adjacent normal epithelium. Quantitative, longitudinal estimates of how expression of MMPs 2, 9, and 14 changes as lobular tissue acquires an increasingly neoplastic phenotype are necessary to further establish the biological plausibility of an association with invasive breast cancer risk. The results of this study will serve as a necessary comparison point for future studies of MMP expression in LCIS with invasive breast cancer.

## Methods

This study was approved by the University of North Carolina (UNC) at Chapel Hill Biomedical Institutional Review Board and was conducted according to the United States Code of Federal Regulations Title 21 (Part 16 – Protection of Human Subjects and Part 56 – Institutional Review Boards) and other applicable regulations and laws. Waivers of informed consent and HIPAA approval were granted for the study of retrospective data.

### Patient population

This cross-sectional retrospective pilot study included women aged ≥18 years who were diagnosed with LCIS at UNC Hospitals between 2004 and 2014. A search of our institutional database identified patients who underwent breast surgery or breast biopsy and whose final diagnosis contained the terms “lobular carcinoma *in situ*” or “lobular neoplasia”. “Lobular neoplasia” was used in the database search to ensure that we identified all potential cases of LCIS. However, lobular neoplasia cases in which no tissue met the histologic criteria of LCIS (i.e., only atypical lobular hyperplasia was present) when reviewed by a pathologist (described below) were not included in the study. Previous and concurrent breast cancer diagnoses were identified by medical record review. Patients with a previous or concurrent diagnosis (within one year of LCIS diagnosis) of invasive breast cancer in the same breast as the LCIS were excluded. A total of 116 cases with LCIS were identified and considered for histopathologic review.

### Histopathologic review

An experienced breast pathologist (TJL) reviewed archival hematoxylin and eosin-stained tissue slides from eligible cases to confirm the LCIS diagnosis and identify LCIS and normal tissue sections for analysis.

Based on existing qualitative estimates of marker mean expression, our target sample size was 60 LCIS cases. We anticipated that this sample size would provide precise quantitative estimates of the marker distributions and allow for comparative analyses to be investigated further in future studies.

Cases were randomly selected in waves for histopathologic slide review from the 116 identified as eligible from the pathology report review. In total, we reviewed the slides of 96 LCIS cases to achieve our pre-determined sample size of at least 60 LCIS cases. From the 96, cases were excluded because: they represented a repeat diagnosis in the same patient (N = 11); pathology reports and/or tissue blocks were unavailable (N = 4); there was a lack of LCIS in the initial sample or on tissue re-cuts (N = 15); or invasive cancer was present (N = 2). Thus, 64 cases were included in this analysis – 16 (25%) were obtained from a core biopsy, 35 (55%) were from excisional/incisional biopsy or lumpectomy, and 13 (20%) were from a mastectomy.

For each case, the pathologist used published criteria to determine the histologic subtype of LCIS represented in more than 50% of the LCIS-involved lobules (classic, florid, or pleomorphic)^4,38–41^. Representative formalin-fixed paraffin-embedded tissue blocks were selected for immunofluorescence staining of MMP2, MMP9, MMP14, and collagen IV. We selected one block containing LCIS and one block containing normal breast glandular tissue for comparison. We chose to use normal tissue from the same person as the comparison tissue (rather than tissue from unrelated, healthy donors) in order to control for patient-level factors that may influence MMP and/or collagen IV levels. A new H&E-stained slide was created to confirm tissue histology and two unstained tissue sections were prepared for analysis.

### Immunofluorescence staining

Sequential dual immunofluorescence staining of MMP2, MMP9, MMP14, and collagen IV was performed on 5 µm-thick formalin-fixed paraffin-embedded whole tissue sections at the UNC Translational Pathology Laboratory (see Fig. 1). MMP2 and MMP14 were evaluated on the same slide; and MMP9 and collagen IV were evaluated on the same slide.

Staining was conducted using the Bond fully-automated slide staining system and Bond Polymer Refine Detection kit # DS9800 (both from Leica Biosystems Inc., Norwell, MA). Individual staining runs included LCIS and normal slides. Slides were deparaffinized and hydrated using Bond dewax and wash solutions. Epitope retrieval for the first primary antibody (to MMP14 or collagen IV; see Table 2) was performed for 20 min at 100 °C in Bond-epitope retrieval solution 1. Epitope retrieval was followed by 5 min of endogenous peroxidase blocking using Bond peroxide blocking solution for HSP40 and a 10 min protein blocking step (PV6122, Leica). Slides were then incubated with the first primary antibody for one hour at room temperature, followed by Bond Polymer (for MMP14) or Bond Post Primary and Polymer (for collagen IV) secondary antibodies and Cy3 tyramide signal amplification (Perkin Elmer, Boston, MA) for visualization.

**Table 2:** Technical details of the primary antibodies used to quantify MMP and collagen IV expression in normal and LCIS breast tissue.

Antibody	Host Clonality	Manufacturer	Catalog #	Dilution
MMP 2	Mouse monoclonal IgG	Millipore/Chemicon (Billerica, MA)	MAB13405	1:50
MMP 9	Rabbit monoclonal IgG	Epitomics/Cell Marque (Rocklin, CA)	AC-0122RUO	1:100
MMP 14	Rabbit polyclonal	Sigma-Aldrich (St. Louis, MO)	HPA051432	1:50
Collagen IV	Mouse monoclonal IgG	Cell Marque/Sigma (Rocklin, CA)	239M-15	1:200

Following staining for the first primary antibody, the second epitope (MMP2 or MMP9) was unmasked using epitope retrieval solution 1 for 20 minutes at 100 °C. This step also served to strip the slides of the primary and secondary antibodies from the first stain. Slides were incubated for one hour at room temperature with the second primary antibody (to MMP2 or MMP9; see Table 2), followed by Bond Post Primary and Polymer (for MMP2) or Bond Polymer (for MMP9) secondary antibodies and Cy5 tyramide signal amplification (Perkin Elmer). A nuclear counterstain was applied to all slides (Hoechst 33258, Life Technologies, Carlsbad, CA) before they were mounted with ProLong Gold Antifade Reagent (Life Technologies).

Normal breast, skin, and spleen tissues were included with each staining run. Assaying of these tissues with the primary and secondary antibodies and the detection system served as a positive control. Assaying of the tissues with only secondary antibodies and the detection system (primary antibody omitted) served as a negative control.

### Automated analysis of immunofluorescence staining

H&E and immunostained slides were scanned to create digital images using the Aperio FL slide scanner (Leica Biosystems) at a magnification of 20X. Regions of LCIS or normal glands were manually annotated by a trained research specialist using ImageScope 12.2 (Leica Biosystems). Annotation accuracy was verified by the pathologist. Definiens Tissue Studio v2.5 and Tissue Studio Library v4.2 (Definiens AG, Munich, Germany) were used to analyze the images. Annotated areas were preselected for region-of-interest detection. Each marker was analyzed separately.

MMP2, MMP9, and MMP14 were evaluated within the manually-annotated LCIS and normal breast epithelial tissue regions. The Definiens Nuclei and Simulated Cells algorithm was used to determine the number of cells positive for each marker in the annotated regions. Staining intensity was classified as negative, low (1+), medium (2+), or high (3+). To define the thresholds for each category, the median of the average staining intensity for each of the MMP markers was determined from a test-set of 14 annotated images that included a mix of LCIS and normal tissue slides, and was used to set the threshold between 1+ and 2+ staining intensities. The negative/1+ and the 2+/3+ intensity thresholds were then set equidistant from the median. These thresholds were used by the analysis algorithm to categorize the staining intensity of each cell as positive or negative and to calculate a histological score (H Score) for all of the analyzed cells in each image (see Supplementary Fig. S3):

$${\rm{H}}\,{\rm{s}}{\rm{c}}{\rm{o}}{\rm{r}}{\rm{e}}=(1\times {\rm{ \% }}\,{\rm{l}}{\rm{o}}{\rm{w}}\,{\rm{i}}{\rm{n}}{\rm{t}}{\rm{e}}{\rm{n}}{\rm{s}}{\rm{i}}{\rm{t}}{\rm{y}}\,{\rm{c}}{\rm{e}}{\rm{l}}{\rm{l}}{\rm{s}}\,[1+])+(2\times {\rm{ \% }}\,{\rm{m}}{\rm{e}}{\rm{d}}{\rm{i}}{\rm{u}}{\rm{m}}\,{\rm{i}}{\rm{n}}{\rm{t}}{\rm{e}}{\rm{n}}{\rm{s}}{\rm{i}}{\rm{t}}{\rm{y}}\,{\rm{c}}{\rm{e}}{\rm{l}}{\rm{l}}{\rm{s}}[2+])+(3\times {\rm{ \% }}\,{\rm{h}}{\rm{i}}{\rm{g}}{\rm{h}}\,{\rm{i}}{\rm{n}}{\rm{t}}{\rm{e}}{\rm{n}}{\rm{s}}{\rm{i}}{\rm{t}}{\rm{y}}\,{\rm{c}}{\rm{e}}{\rm{l}}{\rm{l}}{\rm{s}}[3+])$$

For collagen IV, the Definiens Composer algorithm was used in addition to the manual annotations described above to further segment the tissue image and enrich for the basement membrane area surrounding annotated LCIS and normal epithelial regions. Using the Composer, the percentage of pixels positive for collagen IV was calculated as the number of pixels that stained above background intensity divided by the total number of pixels in the basement membrane region immediately surrounding the lobular unit. The average intensity of collagen IV staining in the basement membrane region was measured on a scale of 0 to 64,000.

### Patient and clinical covariates

Age at surgery, diagnosis year, and biopsy/surgery type were abstracted from pathology reports. Race, parity, age at menarche, smoking status, alcohol use, menopausal hormone use, height, weight, and family history of breast cancer were abstracted from electronic health records and reflect patients’ status within one year of the LCIS surgery. A woman was classified as postmenopausal if she reported that she was no longer experiencing menstrual periods or if she had a prior hysterectomy or bilateral oophorectomy. Menopausal hormones were defined as estrogen and/or progestin being taken by a postmenopausal woman for the relief of menopausal symptoms. BI-RADS®^42^ mammographic density was abstracted from radiology reports dated no more than two years prior to the date of LCIS diagnosis (median − 43 days).

### Statistical analysis

#### Population characteristics

To characterize the study population, we tabulated LCIS characteristics overall and stratified by whether paired normal tissue was available.

#### Quantification of MMP and collagen IV expression

In order to understand the degree to which the MMPs were expressed in LCIS and normal epithelium, we calculated measures of expression prevalence and expression intensity for each. A sample was defined as expressing MMP2, MMP9, or MMP14 if the MMP in question was expressed in ≥10% of the cells within the sample’s manually annotated areas (see *Automated analysis of immunofluorescence staining*, above). The same criteria were used for the evaluation of LCIS and normal tissues. As a sensitivity analysis, we explored how our results would be affected if we used a lower (1%) or higher (20%) threshold for defining expression prevalence. The results were similar to what we observed using the 10% threshold and are not presented here. Staining intensity was classified as 1+, 2+, or 3+, as described above (in *Automated analysis of immunofluorescence staining*). The H score was used as a combined measure of expression prevalence and intensity and was analyzed as a continuous variable.

To evaluate associations between quantitative expression of different markers in a given tissue type, we estimated pairwise correlations, with 95% confidence intervals, among markers in LCIS and in normal tissues. We calculated Spearman rank correlations to account for potential non-normality of distributions.

#### Comparison of MMP and collagen IV expression in LCIS and paired normal breast tissue

The characteristics of cases with and without paired normal tissue were compared using the Fisher exact test (categorical variables) or the Mann-Whitney U test (continuous variables). To evaluate whether expression of a given marker differed in LCIS vs. normal breast epithelium, differences in MMP H score distributions and collagen IV expression were compared between paired LCIS and normal breast samples using the Wilcoxon signed-rank test.

All statistical tests were two-sided and P-values less than 0.05 were considered statistically significant. No formal adjustments for multiple comparisons were made due to the exploratory nature of these analyses. Data were analyzed in SAS v9.4 (SAS, Cary, NC) and are reported in accordance with the REMARK criteria^43^.

## Supplementary information

Supplementary Information

Supplementary Data

## Data Availability

All data analyzed during this study are included in this published article and its supplementary information files (Supplementary Data).
