# Supplementary material for: Quantitative expression of MMPs 2, 9, 14, and collagen IV in LCIS and paired normal breast tissue
Source: Sci Rep. 2019 Sep 17;9:13432. doi: 10.1038/s41598-019-48602-6 (PMC6748975; doi:10.1038/s41598-019-48602-6)
Supplement: Supplementary file 1 — Supplementary Information [file 41598_2019_48602_MOESM1_ESM.pdf]

Supplementary Information File

Title: Quantitative expression of MMPs 2, 9, 14, and collagen IV in LCIS and paired normal breast tissue

Authors: Sarah J. Nyante, Tengteng Wang, Xianming Tan, Emily F. Ozdowski, Thomas J. Lawton

Supplementary Table S1. Characteristics of 64 patients diagnosed with lobular carcinoma *in situ* (LCIS) at a single institution, 2004-14.

Characteristic	Overall (N=64)		Case Has Paired Normal Breast Tissue?				
			Yes (N=44)		No (N=20)		
	N	%	N	%	N	%	P-Value
Age at surgery (years)[a]							
≤ 50	27	42	22	50	5	25	0.10
> 50	37	58	22	50	15	75	
Race							
White	44	70	32	73	12	63	0.55
Nonwhite	19	30	12	27	7	37	
Unknown	1				1		
Menopausal status							
Premenopausal	27	42	20	45	7	35	0.59
Postmenopausal	37	58	24	55	13	65	
Parous							
No	13	21	10	23	3	16	0.74
Yes	50	79	34	77	16	84	
Unknown	1				1		
Age at menarche (years)							
≤ 12	29	54	18	51	11	58	0.78
> 12	25	46	17	49	8	42	
Unknown	10		9		1		
BMI (kg/m^2)[b]							
< 25	20	35	16	41	4	22	0.41
25 to < 30	14	25	9	23	5	28	
≥ 30	23	40	14	36	9	50	
Unknown	7		5		2		
Alcohol use							
None	28	51	17	45	11	65	0.17
Less than once per day	16	29	14	37	2	12	
Once or more per day	11	20	7	18	4	24	

Unknown	9						
Hormone use							
Never	45	73	34	79	11	58	0.21
Former user	8	13	4	9	4	21	
Current user	9	15	5	12	4	21	
Unknown	2						
Chemoprevention use[c]							
No	57	89	38	86	19	95	0.42
Yes	7	11	6	14	1	5	
Family history of breast cancer							
No	49	78	35	80	14	74	0.74
Yes	14	22	9	20	5	26	
Unknown	1				1		
Mammographic density							
Non-dense	23	42	15	41	8	44	1.00
Dense	32	58	22	59	10	56	
Unknown	9		7		2		
Surgery type							
Needle-core biopsy	16	25	1	2	15	75	<0.01
Surgical biopsy/lumpectomy	35	55	31	70	4	20	
Mastectomy	13	20	12	27	1	5	
Histologic Subtype							
Classic	55	86	40	91	15	75	0.22
Florid	7	11	3	7	4	20	
Pleomorphic	2	3	1	2	1	5	

[a]Among all subjects (in years): mean - 53; standard deviation - 9.6; median - 52; range 32 to 76
[b]Among all subjects (in kg/m^2): mean - 28.2; standard deviation - 6.6; median - 27.8; range 16.9 to 45.8
[c]Among all subject: tamoxifen, N=5, anastrozole, N=1; raloxifene, N=1

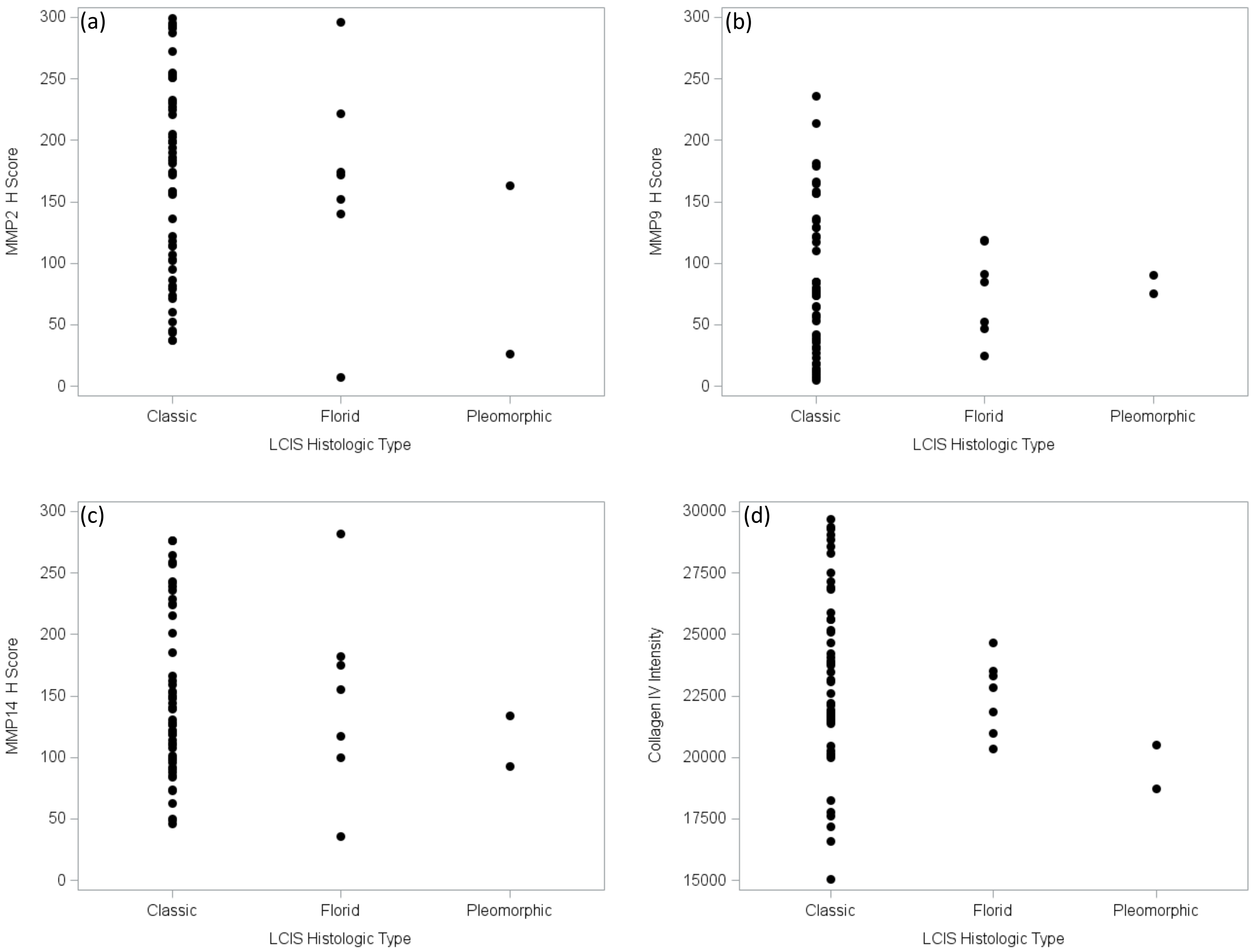

Supplementary Figure S1. Marker distribution according to LCIS histologic subtype. The data shown represent marker expression measured within the LCIS (n=64). For MMP2 (panel a), MMP9 (panel b), MMP14 (panel c), and Collagen IV (panel d), marker distributions in florid LCIS or pleomorphic LCIS did not differ from the distributions in classic LCIS.

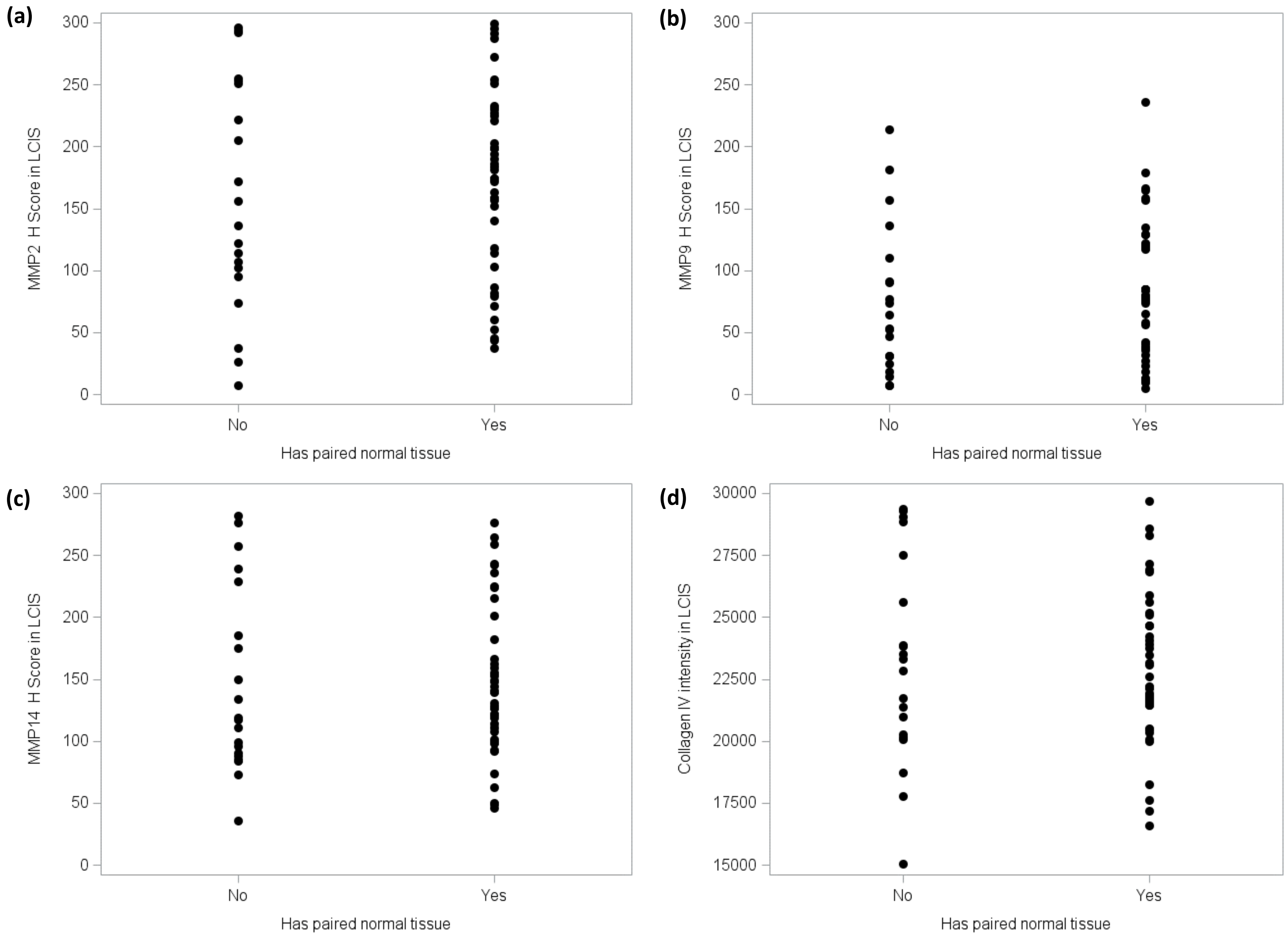

(a)
MMP2 H Score in LCIS
300
250
200
150
100
50
0
No
Yes
Has paired normal tissue
(b)
MMP9 H Score in LCIS
300
250
200
150
100
50
0
No
Yes
Has paired normal tissue
(c)
MMP14 H Score in LCIS
300
250
200
150
100
50
0
No
Yes
Has paired normal tissue
(d)
Collagen IV intensity in LCIS
30000
27500
25000
22500
20000
17500
15000
No
Yes
Has paired normal tissue

(e)	Sample has paired normal tissue?				
Marker expression in LCIS	Yes (N=44)		No (N=20)		Mann-Whitney U test P-Value
	Mean	SD	Mean	SD	
**MMP2 H score**	172	73	161	93	0.62
**MMP9 H score**	81	55	74	59	0.54
**MMP14 H score**	147	60	146	74	0.57
**Collagen IV intensity**	22854	2969	23157	4112	0.95

Supplementary Figure S2. Marker distribution in LCIS according to availability of paired normal tissue. Panels a-d show H score (MMP2, MMP9, MMP14) or expression intensity (collagen IV) distributions stratified by whether the patient had normal breast tissue available for analysis. For each marker, distributions are similar between the two groups. Further, mean values of marker expression did not differ significantly for any of the markers, as shown by the data in panel e.

MMP2

H score difference =29

LCIS

(a)

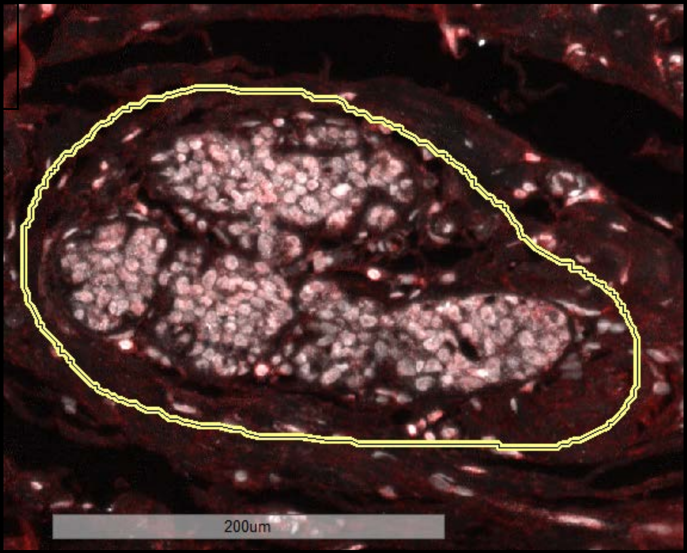

normal epithelium

(b)

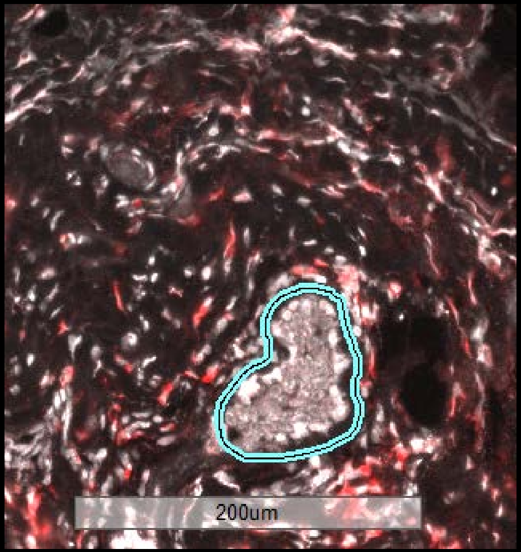

H score difference =-188

LCIS

(c)

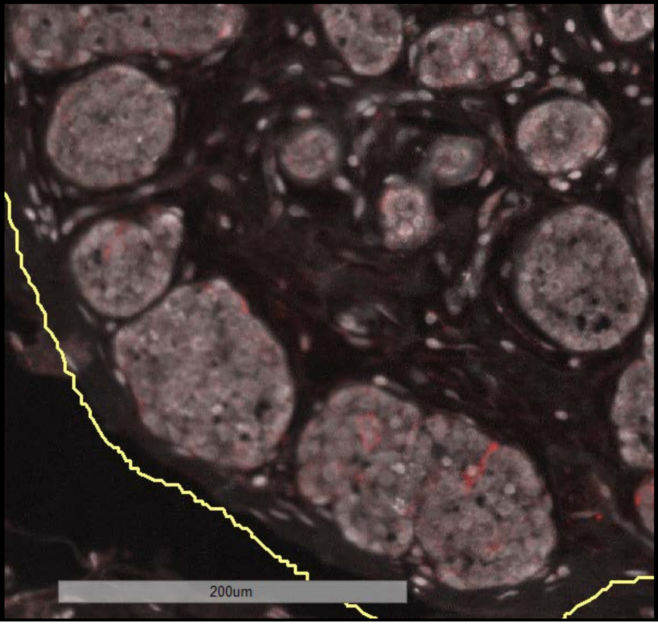

normal epithelium

(d)

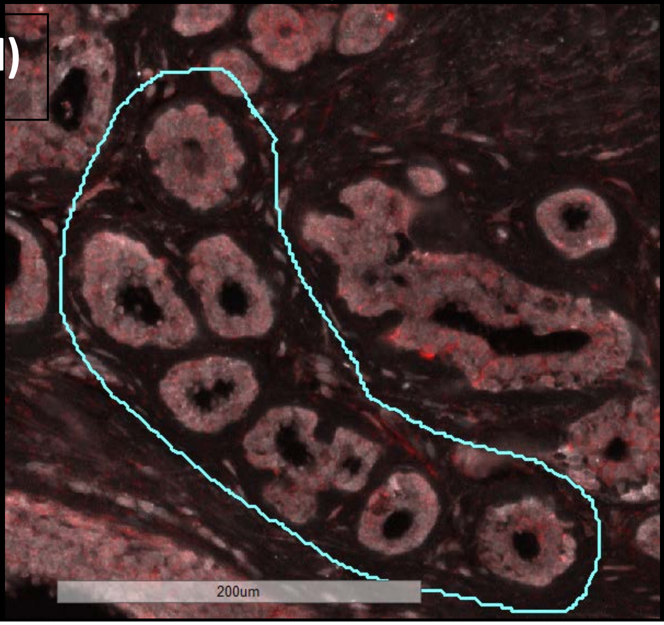

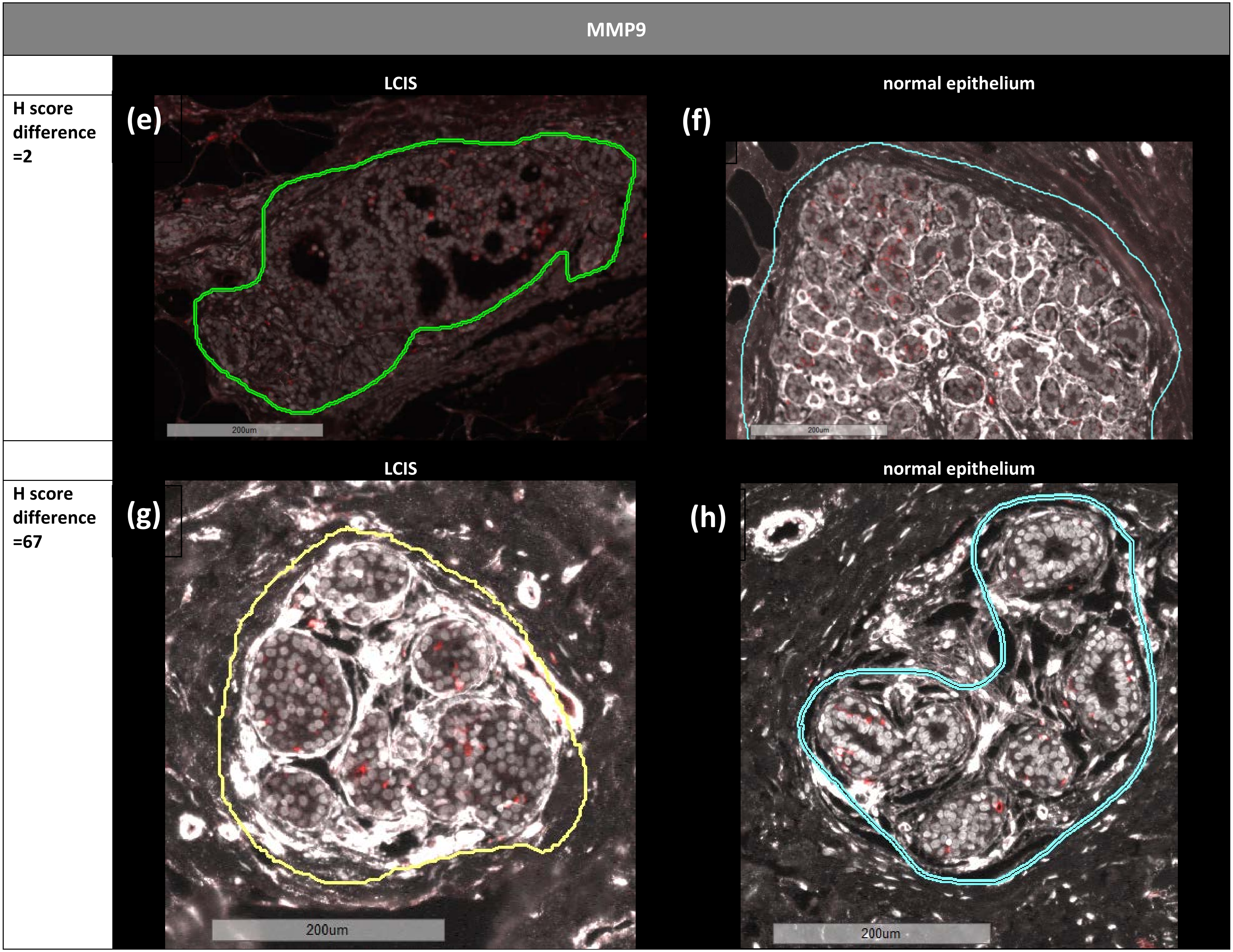
MMP9
LCIS
normal epithelium
H score difference =2
(e)
200um
(f)
200um
LCIS
normal epithelium
H score difference =67
(g)
200um
(h)
200um

MMP14

	LCIS	normal epithelium
H score difference =3	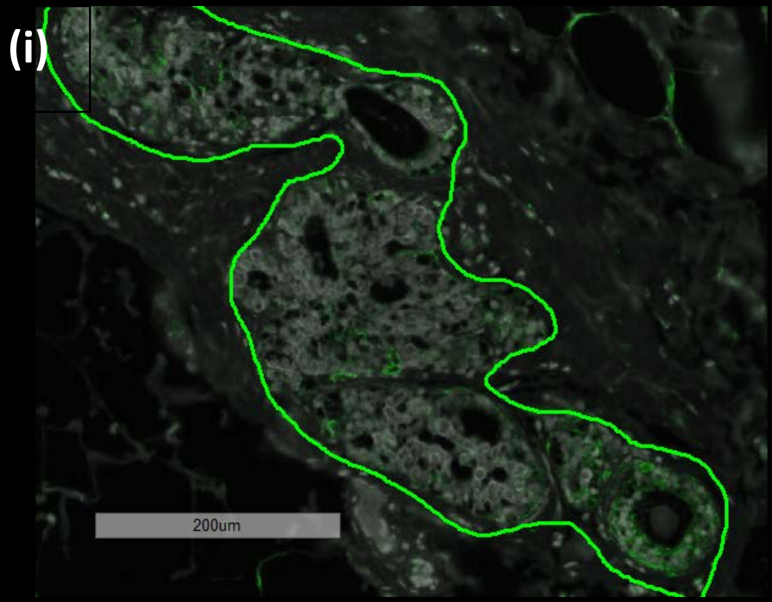 (i) 200um	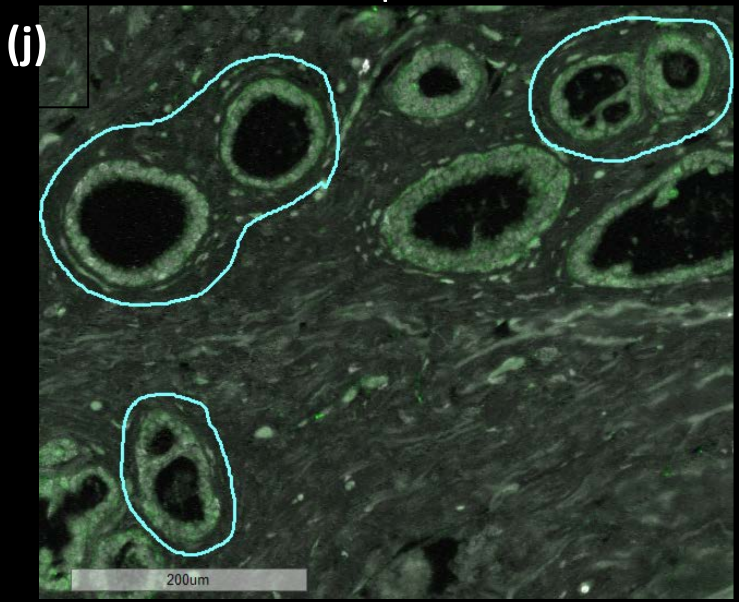 (j) 200um
	LCIS	normal epithelium
H score difference =114	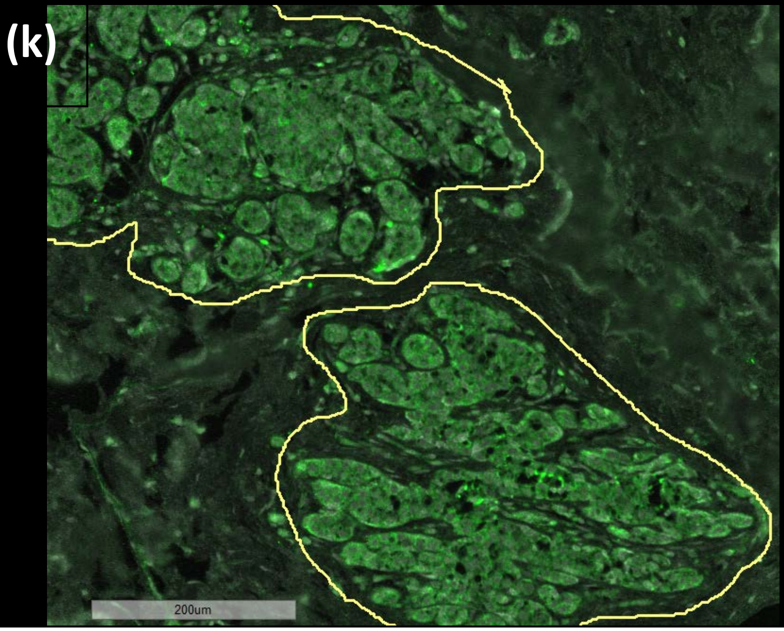 (k) 200um	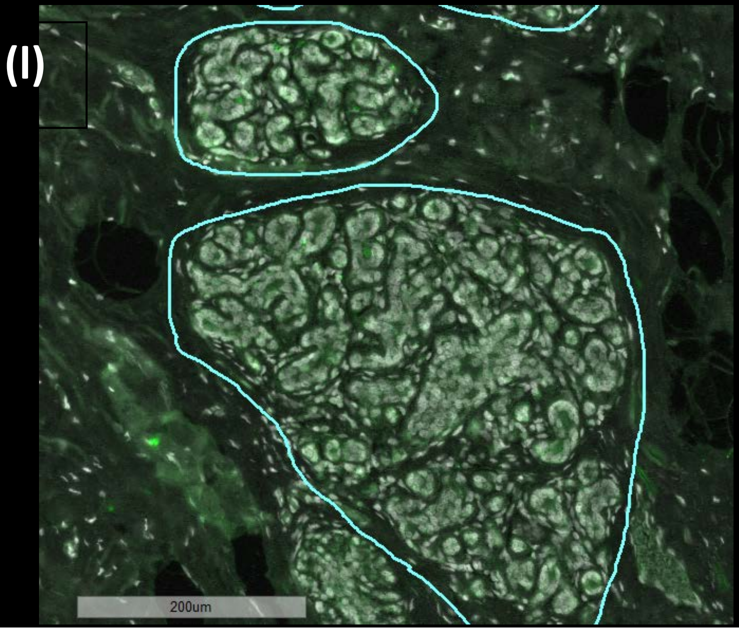 (l) 200um

Collagen IV		
	LCIS	normal epithelium
Intensity difference = -103	(m) 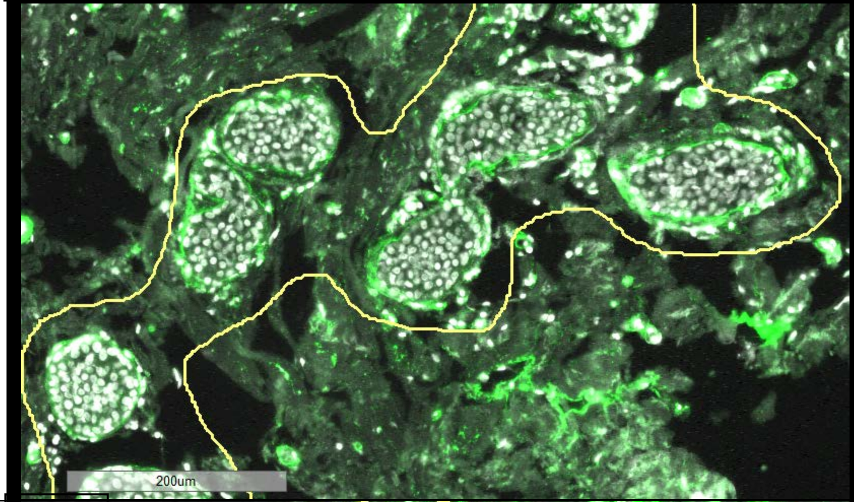	(n) 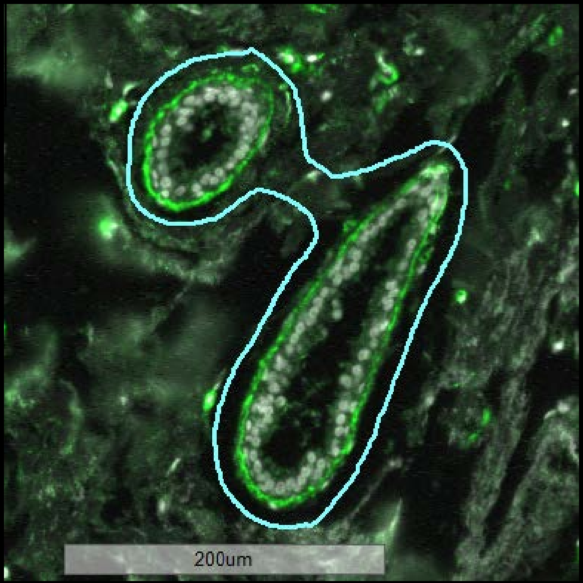
Intensity difference = 9809	(o) 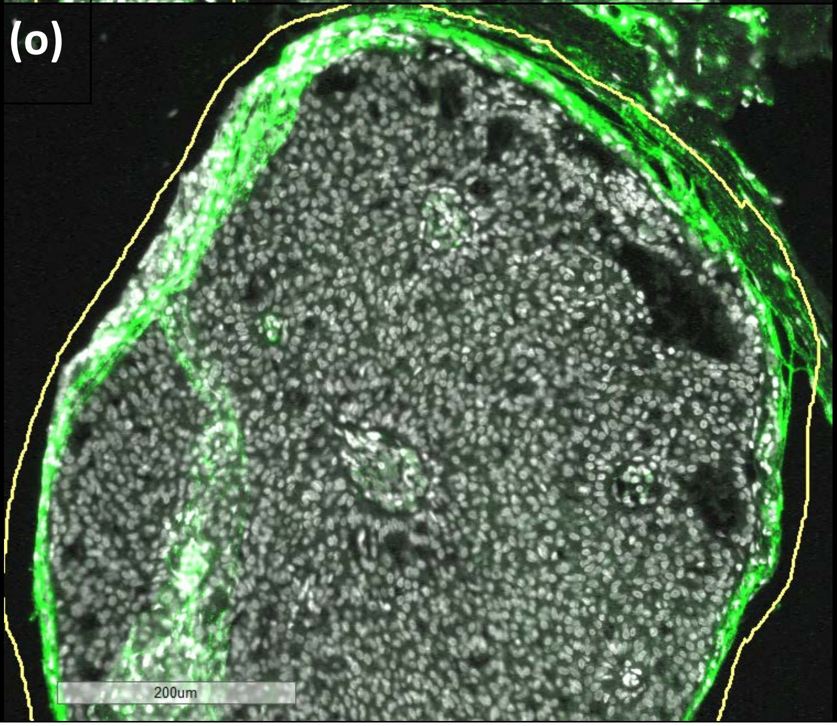	(p) 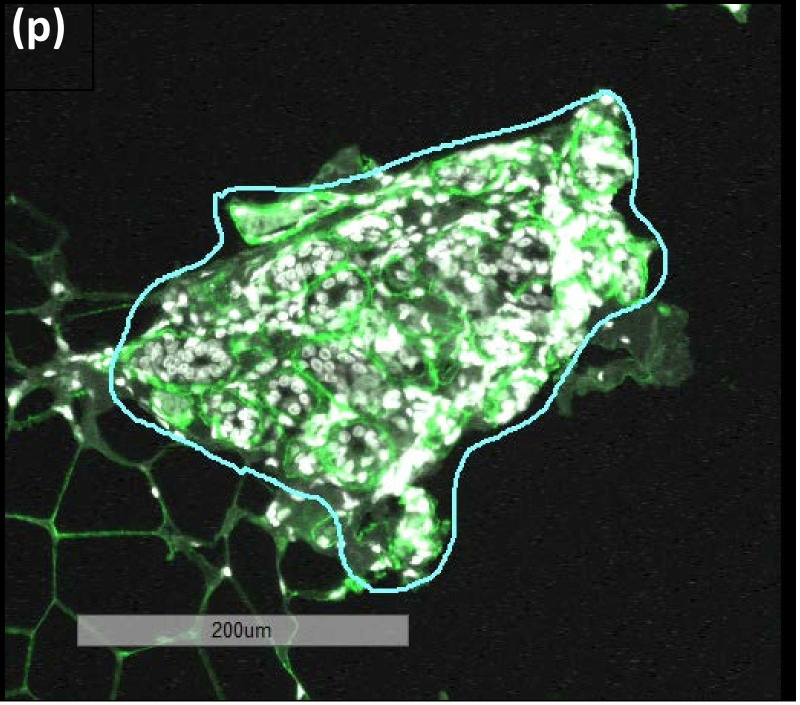

Supplementary Figure S3. Example images of MMP and collagen IV staining in LCIS and paired normal breast tissue. Examples of concordant (*i.e.*, low difference in H scores) and discordant (*i.e.*, high difference in H scores) levels of MMP and collagen IV expression are shown above. Panels a-d show representative staining for MMP2; panels e-h show representative staining for MMP9; panels i-l show representative staining for MMP14; and panels m-p show representative staining for collagen IV. The images shown are examples of representative areas. However, the H score and intensity (and therefore the differences) are calculated based on regions of interest (circled in yellow [LCIS] or blue [normal]) across the whole slide.
